# Paternal preconceptional metformin exposure induces metabolic dysregulation in offspring

**DOI:** 10.1038/s41421-026-00913-5

**Published:** 2026-07-01

**Authors:** Tao Pan, Guangyuan Fan, Xingyu Ji, Yuqing Zhou, Yuan Li, Feifei Wang, Changyou Jiang, Xing Liu, Lan Ma, Qiumin Le

**Affiliations:** 1 State Key Laboratory of Medical Neurobiology, School of Basic Medical Sciences, MOE Frontiers Center for Brain Science, Institutes of Brain Science, Fudan University, Shanghai, China; 2 Research Unit of Addiction Memory, Chinese Academy of Medical Sciences (2021RU009), Shanghai, China

**Keywords:** Epigenetics, Metabolomics

Dear Editor,

Metformin, the first-line therapy for type 2 diabetes^1,2^, is increasingly used not only in diabetic patients but also being actively explored for cardiovascular risk reduction, anti-neoplastic effects, with real-world adoptions in healthy individuals for metabolic risk management and healthy aging^3–5^. While the Paternal Origins of Health and Disease (POHaD) paradigm posits that preconceptional environmental exposures in fathers can reprogram offspring health through epigenetic modifications in sperm^6–9^, the intergenerational consequences of paternal metformin use remain elusive. Recent clinical debates have focused on whether paternal metformin exposure increases the risk of genital malformations in male offspring; however, findings remain conflicting, with a Danish registry study reporting a positive association^10^ while larger multinational cohorts found no such link^11^. Crucially, against the backdrop of the POHaD paradigm, beyond the risk of congenital malformations, the intergenerational impact of paternal metformin use on offspring metabolism, particularly via epigenetic mechanisms, remains unknown.

To investigate the transgenerational impact of paternal metformin treatment, we administered metformin (Met, 200 mg/kg) or vehicle (Veh, equivalent volume of saline) to 8-week-old male Sprague-Dawley rats for 21 days and mated them with untreated females to generate offspring (Fig. 1a). We observed no differences in litter size, sex ratio, or birth weight between the Met-F1 and Veh-F1 groups (Supplementary Fig. S1a–c). However, Met-F1 offspring of both sexes exhibited significantly higher body weight since week 8, compared to Veh-F1 offspring (Fig. 1b, left). By week 15, Met‑F1 offspring exhibited a significant right shift in body weight *Z*‑score distribution. The incidence of overweight (*Z*‑score ≥ 2) reached 50.0% in males and 45.8% in females, compared to only 4.2% and 0.0% in Veh‑F1 males and females, respectively (Fig. 1b, right). MRI-based body composition analysis confirmed that this increased body weight in both sexes was due to a specific increase in fat mass, with no change in lean mass (Fig. 1c).

**Fig. 1:**
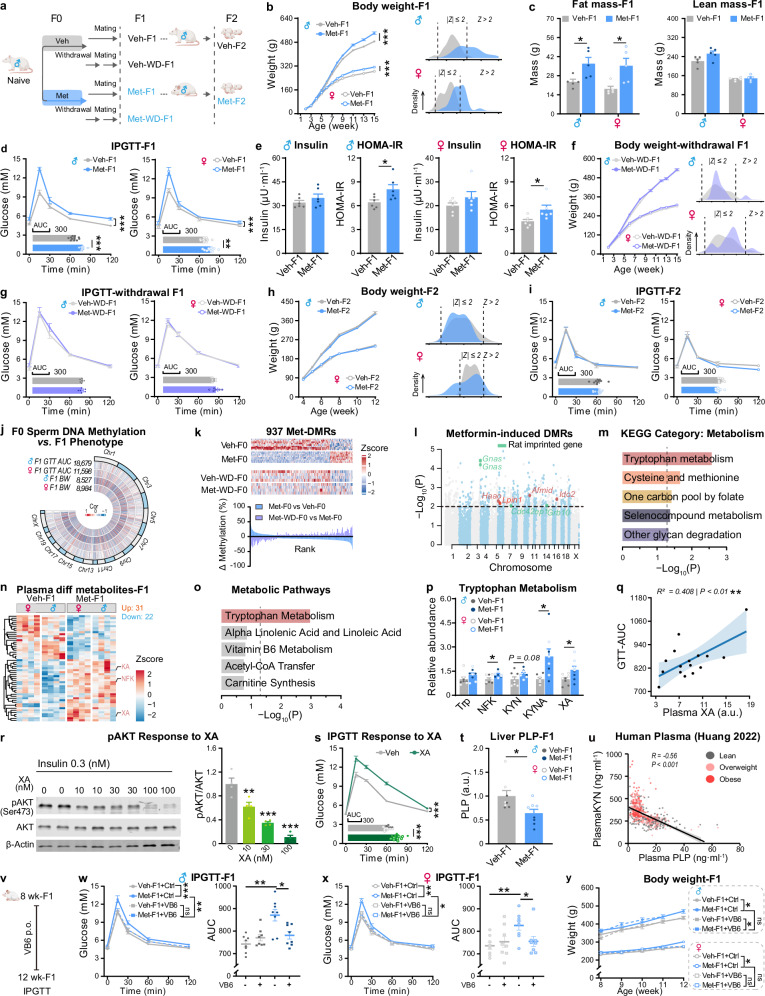
Paternal metformin induces intergenerational metabolic dysfunction in F1 offspring. **a** Schematic of the experimental design for paternal metformin treatment, drug washout, and transgenerational transmission. **b** Left, body weight of F1 male and female offspring (Veh-F1, *n* = 24 from 12 litters; Met-F1, *n* = 24 from 12 litters). Right, distribution of body weight *Z*‑scores at 15 weeks, with density curves colored by *Z*‑score values. **c** MRI-based quantification of fat mass (left) and lean mass (right) in F1 male and female offspring (Veh-F1, *n* = 5 from 5 litters; Met-F1, *n* = 5 from 5 litters). **d** IPGTT curves and corresponding AUC for F1 male and female offspring (Veh-F1, *n* = 12 from 12 litters; Met-F1, *n* = 12 from 12 litters). **e** Fasting insulin levels and HOMA-IR in F1 male and female offspring (Veh-F1, *n* = 6 from 6 litters; Met-F1, *n* = 6 from 6 litters). **f** Left, body weight of F1 male and female offspring generated after 3-week drug withdrawal (Veh-WD-F1, *n* = 12 from 6 litters; Met-WD-F1, *n* = 12 from 6 litters). Right, distribution of body weight at 15 weeks, with density curves shaded by *Z*-scores. **g** IPGTT curves and AUC for F1 male and female offspring generated after 3-week drug withdrawal (Veh-WD-F1, *n* = 6 from 6 litters; Met-WD-F1, *n* = 6 from 6 litters). **h** Left, body weight of F2 male and female offspring (Veh-F2, *n* = 10 from 5 litters; Met-F2, *n* = 12 from 6 litters). Right, distribution of body weight at 12 weeks, with density curves shaded by *Z*-scores. **i** IPGTT curves and AUC for F2 male and female offspring (males: Veh-F2, *n* = 10 from 10 litters, Met-F2, *n* = 11 from 11 litters; females: Veh-F2, *n* = 7 from 7 litters, Met-F2, *n* = 10 from 10 litters). **j** Circular heatmap showing correlations between F0 sperm DNA methylation and body weight (BW) or glucose tolerance (GTT AUC) in male and female F1 offspring. **k** Upper, heatmap of metformin-induced differentially methylated regions (DMRs) in F0 sperm. Lower, ranking plot of methylation changes at metformin-associated DMRs across two comparisons: Met-F0 vs Veh-F0 (metformin effect), Met-WD-F0 vs Met-F0 (withdrawal effect). **l** Chromosomal distribution of metformin-induced DMRs. Regions associated with rat imprinted genes (green) or the tryptophan pathway (red) are highlighted. **m** KEGG pathway enrichment analysis of genes overlapping metformin-induced DMRs. **n** Heatmap of 53 significantly altered metabolites identified by untargeted plasma metabolomics in F1 offspring at 17 weeks (males: Veh-F1, *n* = 4 from 4 litters, Met-F1, *n* = 4 from 4 litters; females: Veh-F1, *n* = 4 from 4 litters, Met-F1, *n* = 4 from 4 litters). Selection criteria: VIP > 1, *P* < 0.05, and fold change > 1.2 or < 0.83. **o** Metabolic pathway enrichment of differential metabolites. **p** Relative abundance of tryptophan pathway intermediates in F1 plasma, presented as a bar chart (*n* as in **n**). **q** Linear regression analyses between plasma XA concentrations and glucose tolerance (GTT AUC) in F1 offspring. **r** Insulin signaling in primary skeletal muscle cells treated with increasing doses of XA (0 nM, 10 nM, 30 nM, 100 nM). Left: representative western blots for phosphorylated AKT (Ser473), total AKT, and β-actin following acute insulin stimulation (0.3 nM); Right: quantification of pAKT/AKT ratio (*n* = 4 independent cultures per condition). **s** Acute effects of exogenous XA (100 mg/kg, i.p.) on systemic glucose homeostasis in naïve Sprague-Dawley rats (*n* = 16 per group). **t** Liver pyridoxal 5′-phosphate (PLP) levels in F1 offspring (*n* as in **n**). **u** Plasma PLP and kynurenine (KYN) concentrations across human BMI categories (lean: BMI 18–24, *n* = 354; overweight: BMI 24–28, *n* = 268; obese: BMI ≥ 28, *n* = 113) (data from Huang et al.^14^). **v** Schematic of vitamin B6 (VB6) treatment protocol. **w**, **x** IPGTT curves and AUC in male (**w**) and female (**x**) F1 offspring after VB6 treatment (Veh-F1 + Ctrl, *n* = 8 from 8 litters; Met-F1 + Ctrl, *n* = 8 from 8 litters; Veh-F1 + VB6, *n* = 8 from 8 litters; Met-F1 + VB6, *n* = 8 from 8 litters). **y** Body weight trajectories of F1 male and female offspring during the 4-week VB6 treatment (*n* as in **w**). Data are presented as mean ± SEM. Significance thresholds: *P < 0.05, **P < 0.01, ***P < 0.001.

This adiposity phenotype was accompanied by increased organ index of gonadal white adipose tissue (gWAT) and liver (Supplementary Fig. S2a–c, f–h). In contrast, the organ index of inguinal white adipose tissue (iWAT) remained unchanged, whereas a female-specific increase in that of brown adipose tissue (BAT) was observed (Supplementary Fig. S2d, e, i, j). Histological analysis revealed adipocyte hypertrophy in gWAT and iWAT of both sexes (Supplementary Fig. S2k–m, q–s). Specifically in male Met-F1 offspring, we also observed whitening of BAT and the development of hepatic steatosis, with the latter quantified by a significant increase in Oil Red O^+^ area (Supplementary Fig. S2n–p, t–v). Moreover, male Met-F1 offspring developed dyslipidemia (elevated triglycerides, low-density lipoprotein cholesterol (LDL-C), and high-density lipoprotein cholesterol (HDL-C)), while females showed elevated triglycerides and LDL-C but normal HDL-C (Supplementary Fig. S3a–h). Met-F1 offspring showed reduced dark-phase locomotor activity, despite unchanged food intake and respiratory quotient, indicating decreased energy expenditure (Supplementary Fig. S4a–f).

We then assessed glucose metabolism. Both male and female Met-F1 offspring developed glucose intolerance, as shown by significantly elevated blood glucose levels during an intraperitoneal glucose tolerance test (IPGTT) and an increased area under the curve (AUC) (Fig. 1d). While the intraperitoneal insulin tolerance test (IPITT) showed no difference in overall AUC between groups, male Met-F1 exhibited higher blood glucose levels at the 120-min time point, indicating impaired insulin responsiveness or clearance kinetics. In contrast, female Met-F1 offspring showed no such deviation at any time point (Supplementary Fig. S5a, b). Nevertheless, both male and female offspring displayed elevated homeostatic model assessment for insulin resistance (HOMA-IR), driven by fasting hyperglycemia in the absence of changes in insulin level (Fig. 1e). To define the tissue-specific basis of this metabolic phenotype, we analyzed in vivo insulin-stimulated AKT phosphorylation and found significantly attenuated phospho-AKT levels in skeletal muscle (but not liver) of Met-F1 offspring (Supplementary Fig. S5c, d), indicating muscle-specific impairment of insulin signaling.

Given that paternal metformin exposure is an acquired pharmacological intervention, we next asked whether its reprogramming of offspring metabolism is reversible or persists after drug cessation. To test this, we introduced a 3-week washout period prior to mating (Fig. 1a). This discontinuation of treatment abolished the adverse metabolic phenotypes in F1 offspring. Offspring from the withdrawal cohort (Met-WD-F1) exhibited normalized body weight trajectories. Throughout the observation period, both male and female Met-WD-F1 offspring showed no significant differences in body weight compared to controls (Veh-WD-F1) (Fig. 1f, left). At week 15, the distribution of body weight *Z*-scores in Met-WD-F1 offspring did not deviate significantly from that of controls (Fig. 1f, right). Furthermore, both male and female Met-WD-F1 offspring displayed glucose tolerance comparable to controls, with no significant differences in blood glucose levels or AUC during IPGTT (Fig. 1g). Bivariate phenotypic analysis confirmed that the overall metabolic phenotype of offspring from the withdrawal cohort closely resembled that of controls, with geometric distances consistently below 2 (Supplementary Fig. S6a, b). These findings indicate that the observed intergenerational effects require sustained drug exposure until conception and are not permanently imprinted. In addition, we investigated whether the intergenerational effects of metformin depend on the paternal metabolic state. To this end, we induced paternal obesity in rats using a high-fat diet (HFD) and evaluated the impact of metformin treatment in this context on offspring body weight trajectories and glucose tolerance (Supplementary Fig. S7a–h, detailed in Supplementary Results and Discussions).

We also investigated whether the metabolic abnormalities observed in F1 offspring could be transmitted to the F2 generation through the paternal lineage. In contrast to the F1 offspring, the F2 offspring showed no significant differences in body weight (Fig. 1h, left). By week 12, both male and female Met-F2 rats displayed body weight *Z*-score distributions comparable to those of the control group (Fig. 1h, right). Furthermore, IPGTT and IPITT revealed no discernible impairments in glucose or insulin sensitivity in either sex relative to controls (Fig. 1i, o; Supplementary Fig. S8a, b). These findings indicate that the metabolic dysfunctions induced by paternal metformin exposure are not stably transmitted beyond the F1 generation.

Paternal intergenerational epigenetic inheritance is mediated by multiple sperm-borne factors, including small non-coding RNAs, DNA methylation, and histone modifications. Among these, although the sperm non-coding RNA mechanisms are well established, we observed robust, reversible, and phenotype-associated changes in DNA sperm methylation.

We performed reduced representation bisulfite sequencing (RRBS) on F0 sperm from all six experimental groups (Veh, Met, Veh-WD, Met-WD, and for HFD-Veh, HFD-Met in Supplementary text), capturing a total of 276,842 methylated regions (Supplementary Fig. S9a). Unsupervised hierarchical clustering revealed that the sperm methylome was shaped by both paternal diet and metformin exposure (Supplementary Fig. S9b). For the four groups under normal diet, correlation analyses between sperm methylation levels and F1 core metabolic phenotypes (body weight, GTT AUC; Fig. 1j) identified phenotype-linked candidate loci (*P* < 0.05; Supplementary Fig. S9c–f), which were then stratified by paternal metformin exposure to reveal 937 metformin-induced differentially methylated regions (Met-DMRs) linked to 479 genes (Fig. 1k).

Notably, changed methylation levels at Met-DMRs were largely restored after the 3-week preconception washout period (Fig. 1k), providing an epigenetic basis for the phenotypic rescue observed. These Met-DMRs included canonical imprinting control regions (at *Gnas* and *Grb10*) but were predominantly non-imprinted (Fig. 1l). KEGG enrichment analysis highlighted significant enrichment in tryptophan metabolism (Fig. 1m). Integrative analysis with F1 skeletal muscle transcriptomes showed limited overlap (48/479 genes) between Met-DMR-associated genes and differentially expressed genes, with muscle transcriptional changes primarily enriched in glycerolipid metabolism pathways. However, we identified potential transcriptional regulatory relationships, such as altered expression of downstream targets of *Lpin1* (a Met-DMR-associated gene), which warrants further mechanistic investigation (Supplementary Fig. S10a–f).

Consistent with our sperm methylome findings, untargeted plasma metabolomic profiling of Met-F1 offspring (Supplementary Fig. S11a) identified 53 significantly altered metabolites (31 upregulated, 22 downregulated) (Fig. 1n), with pathway enrichment confirming significant perturbations in tryptophan metabolism (Fig. 1o) and selective upregulation of the kynurenine branch, marked by elevated levels of *N*-formylkynurenine (NFK), kynurenic acid (KYNA), and xanthurenic acid (XA) (Fig. 1p). Among these kynurenine metabolites, XA exhibited the strongest positive correlation with GTT AUC across all F1 groups (Fig. 1q), while upstream (NFK, KYN) and the alternative branch product (KYNA) showed no significant association with glucose tolerance (Supplementary Fig. S11b–d). Functional validation further confirmed that XA dose-dependently suppressed insulin-stimulated AKT phosphorylation in primary skeletal muscle cells (Fig. 1r) and acutely impaired glucose tolerance in both male and female naïve rats (Fig. 1s), establishing XA as a direct contributor to impaired glucose homeostasis in Met-F1 offspring.

We next investigated the potential molecular basis for XA accumulation. Pyridoxal‑5’‑phosphate (PLP), the active form of vitamin B6, is an essential cofactor for three key enzymes in the kynurenine pathway: kynureninase, kynurenine aminotransferase, and 3-hydroxykynurenine aminotransferase. Reduced hepatic PLP availability in Met‑F1 offspring (Fig. 1t) coincides with preferentially impaired kynureninase activity that diverts metabolic flux away from XA synthesis^12,13^ (Supplementary Fig. S11e). Consistent with these animal findings, independent reanalysis of published human plasma metabolomic data revealed that circulating PLP levels were inversely correlated with BMI and KYN concentrations^14^ (Fig. 1u; Supplementary Fig. S11f), supporting a conserved role for PLP in regulating kynurenine pathway flux.

We therefore hypothesized that dietary vitamin B6 supplementation could alleviate this cofactor limitation and restore metabolic balance. In an independent cohort, F1 offspring received vitamin B6 in water for 4 weeks, starting at 8 weeks of age (Fig. 1v). This intervention normalized tryptophan-kynurenine metabolite profiles and significantly reduced plasma XA levels (Supplementary Fig. S11g). Critically, vitamin B6 fully rescued glucose intolerance in both sexes (Fig. 1w, x) and restored insulin-stimulated AKT phosphorylation in skeletal muscle (Supplementary Fig. S11h, i). However, body weight trajectories did not differ between vitamin B6‑treated and control offspring (Fig. 1y), indicating that the metabolic benefits of vitamin B6 primarily lie in glucose homeostasis rather than changes in adiposity.

Collectively, these findings provide converging evidence linking paternal metformin exposure to offspring metabolic dysfunction via altered sperm DNA methylation, hepatic PLP deficiency, and subsequent XA accumulation. We demonstrate that vitamin B6 supplementation effectively improves glucose homeostasis in affected offspring by correcting this underlying cofactor deficiency in kynurenine metabolism, representing a potential nutritional intervention to mitigate adverse intergenerational outcomes.

Our study also raises several questions for future investigation. The critical window of paternal metformin exposure that mediates these intergenerational effects remains to be precisely defined, as does the persistence of these effects following longer-term drug use. Further mechanistic studies are needed to elucidate how sperm epigenetic alterations regulate hepatic PLP metabolism in offspring, to explore the potential contribution of other sperm-borne epigenetic carriers, and to investigate the molecular basis of the observed sex differences in metabolic phenotypes. Overall, our findings challenge the prevailing assumption of universal preconceptional metformin safety and highlight the need for careful consideration of paternal medication use in reproductive health planning.

## Supplementary information

Supplementary information

Supplementary Dataset 1

Supplementary Dataset 2

## Data Availability

All data and statistics are provided in Supplementary Dataset 1. Raw NGS data are deposited in the Genome Sequence Archive (GSA) under the accession numbers CRA035150 and CRA035129.
