# Supplementary material for: Paternal preconceptional metformin exposure induces metabolic dysregulation in offspring
Source: Cell Discov. 2026 Jul 1;12:48. doi: 10.1038/s41421-026-00913-5 (PMC13320179; doi:10.1038/s41421-026-00913-5)
Supplement: Supplementary file 1 — Supplementary information [file 41421_2026_913_MOESM1_ESM.pdf]

Supplementary Information for

Paternal Preconceptional Metformin Exposure Induces Metabolic Dysregulation in Offspring

Tao Pan[1,†], Guangyuan Fan[1,†], Xingyu Ji[1], Yuqing Zhou[1], Yuan Li[1], Feifei Wang[1,2], Changyou Jiang[1,2], Xing Liu[1,2,*], Lan Ma[1,2,*] and Qiumin Le[1,2,*]

This file includes:

Supplementary results and discussions

Materials and Methods

Figs. S1 to S12

References (1-30)

Supplementary results

Paternal metformin exposure exerts intergenerational metabolic effects dependent on paternal diet.

We investigated whether the intergenerational effects depend on the paternal metabolic state by applying a 6-week high-fat diet (HFD) or control diet (CND) to naïve SD rats beginning at 4 weeks of age. The rats were then treated with metformin or vehicle while maintained on their respective diets and subsequently mated to generate F1 offspring (Supplementary Fig. S7a). Paternal HFD alone (HFD-Veh) resulted in significantly increased body weight and impaired glucose tolerance in male F1 offspring compared with CND-Veh (Supplementary Figs. S7b, S7d). In female F1 offspring, HFD-Veh showed a near-significant trend toward increased body weight relative to CND-Veh, but no significant differences in glucose tolerance were observed between these groups (Supplementary Figs. S7c, S7e). This sexual dimorphism is consistent with previous reports demonstrating that male offspring are generally more susceptible to paternal diet-induced metabolic disturbances.

Notably, paternal metformin exerted diametrically opposite intergenerational effects depending on the paternal dietary background. Under CND conditions, paternal metformin exposure (CND-Met) induced significant increases in body weight and impaired glucose tolerance in offspring. In contrast, F1 offspring from fathers treated with metformin under HFD conditions (HFD-Met) displayed body weight and glucose tolerance levels intermediate between those of the HFD-Veh and CND-Veh groups, partially rescuing the HFD-induced metabolic dysfunction of offspring. Effect-size analyses quantitatively confirmed that the direction and magnitude of metformin's intergenerational effects were modulated by the paternal diet (Supplementary Fig. S8f). Specifically, under CND conditions, paternal metformin exposure exerted large positive effects on F1 offspring body weight and IPGTT-AUC (Cohen's $d > 0.8$). Under HFD conditions, by contrast, metformin treatment induced large negative effects on body weight and IPGTT-AUC in male

F1 offspring (Cohen's d < –0.8), whereas small negative effects were observed in female F1 offspring (–0.8 < Cohen's d < 0) (Supplementary Fig. S7f).

To integrate these phenotypes, we used bivariate analysis by plotting standardized body-weight against glucose-tolerance Z-scores (Supplementary Figs. S7g, S7h). In offspring of CND-fed fathers, metformin (CND-Met) induced a significant adverse displacement from the CND-Veh baseline (males: 2.50; females: 2.31). In contrast, in offspring of HFD-fed fathers, metformin (HFD-Met) substantially corrected the HFD-induced displacement, moving progeny toward the CND-Veh cluster (males: 1.20; females: 0.42). Strikingly, the resulting HFD-Met and CND-Met clusters were positioned close to each other (males: 0.35; females: 0.42) and lay intermediate between the CND-Veh and HFD-Veh clusters (Supplementary Figs. S7g, S7h). This geometric pattern demonstrates that metformin exerted opposing, diet-dependent effects. Notably, this bivariate analytical approach also confirmed the complete reversibility of metformin's intergenerational effects after drug withdrawal for 3 weeks. Bivariate analysis of the withdrawal cohort showed that the overall metabolic phenotype of Met-WD-F1 offspring closely resembled that of Veh-WD-F1 controls, with a geometric distance of less than 2 between the two groups (Supplementary Figs. S6a, S6b). These findings demonstrate that body weight and glucose tolerance are highly sensitive biomarkers for assessing the intergenerational effects of paternal metformin exposure and their reversibility.

Paternal metformin exposure remodels sperm DNA methylation signatures

In parallel with Met-DMRs, we investigated DNA methylation alterations associated with paternal diet (Supplementary Fig. S9g). In total, we identified 2018 HFD-induced differentially methylated regions (HFD-DMRs) (Supplementary Fig. S9g), which represents a larger number than Met-DMRs (937), indicating strong effect of HFD on sperm methylation remodeling. KEGG pathway enrichment analysis of genes associated with HFD-DMRs revealed significant involvement in arginine biosynthesis and arachidonic acid metabolism (Supplementary Fig. S9h). Cross-comparison analysis revealed a striking reciprocal relationship between the methylation changes induced by HFD and metformin. At HFD-DMRs, metformin treatment under HFD conditions largely reversed the HFD-induced methylation alterations

(Supplementary Fig. S9i). Conversely, under a control diet, metformin administration (CND-Met vs. CND-Veh) elicited methylation changes that closely resembled those caused by HFD alone (Supplementary Fig. S9i). This remarkable reversibility of sperm DNA methylation changes precisely parallels the reversibility of the offspring metabolic phenotypes, providing strong independent evidence that DNA methylation mediates the intergenerational effects of paternal metformin.

Discussions

Our study demonstrates that paternal exposure to metformin induces multidimensional intergenerational metabolic dysfunction in unexposed F1 offspring, encompassing increased adiposity, glucose intolerance, insulin resistance, and dyslipidemia. Discontinuation of metformin three weeks prior to conception largely abrogated adverse offspring phenotypes, coinciding with near-complete restoration of the sperm methylome. This temporal reversibility highlights the remarkable plasticity of the epididymal sperm epigenome and suggests a critical window for intervention. Notably, adverse metabolic phenotypes observed in the F1 generation did not persist in the F2 cohort, which was derived exclusively via the paternal lineage (F0♂ → F1♂ → F2). Since no significant differences in body weight were detected in F2, we did not perform additional body composition analyses in the current study. We acknowledge this limitation and note that the F2 female body weight showed greater variability, which may reflect subtle phenotypic differences requiring further study.

A key and unexpected finding of this study is the diet-dependent bidirectional nature of metformin's intergenerational effects. Under normal dietary conditions, paternal metformin exposure exerted detrimental effects on offspring metabolism, whereas under high-fat diet conditions, it partially protected against HFD-induced metabolic dysfunction. This striking pattern suggests that metformin may act as a "metabolic normalizer" rather than a uniformly beneficial or harmful agent, buffering extreme deviations in paternal metabolic status.

While the protective effect in the HFD context is biologically intriguing, it remains an exploratory observation. We hypothesize that metformin exerts a threshold-dependent effect on the sperm epigenome,

with the direction of effect determined by the father's pre-existing epigenetic state. This "epigenetic set-point" hypothesis is supported by our methylome data, which show that metformin reverses HFD-induced methylation changes at HFD-DMRs while inducing novel changes at Met-DMRs in normal diet fathers. Importantly, this bidirectional effect raises critical questions about the "metabolic balance point" where metformin's intergenerational effects transition from beneficial to harmful. We currently do not know where this threshold lies along the spectrum of paternal metabolic health, nor how it is modulated by dose or treatment duration. Future studies testing a gradient of paternal metabolic states and clinically relevant metformin doses will be required to precisely define this balance point.

In this study, paternal metformin exposure generally elicited comparable effects in male and female offspring across most phenotypic traits; however, sex-specific differences were observed in a limited number of outcomes. For instance, under HFD conditions, metformin treatment exerted large negative effects on body weight and IPGTT-AUC in male F1 offspring (Cohen's $d < -0.8$), whereas only modest negative effects were observed in females ($-0.8 < d < 0$) (Supplementary Fig. S7f). We attribute this sexual dimorphism to the fact that paternal HFD alone induced significantly greater increases in body weight and more severe glucose intolerance in male than in female F1 offspring relative to the control group (CND-Veh) (Supplementary Figs. S7g, S7h). Notably, the heightened susceptibility of male offspring to HFD-induced metabolic disturbances aligns with previous reports demonstrating that paternal environmental exposures generally exert stronger effects on metabolic phenotypes in male than in female progeny[1,2]. In addition, male CND-Met F1 offspring exhibited more severe hepatic lipid accumulation and greater suppression of locomotor activity relative to their female counterparts. From a genetic-epigenetic perspective, such sex-specific phenotypic divergence may arise from sex-dependent regulation governing the recognition and functional interpretation of paternally inherited epigenetic information during embryonic development. Previous studies have demonstrated that paternally derived methylation imprints undergo divergent reprogramming trajectories in male and female embryos following fertilisation[3]: male embryos tend to retain higher levels of paternal methylation signatures, whereas female embryos exhibit more extensive erasure and remodeling of these marks. This sex-biased epigenetic reprogramming may

constitute a key mechanistic basis underlying sexually dimorphic metabolic phenotypes in progeny. Notably, despite these quantitative differences, the core mechanistic pathway involving PLP deficiency and XA accumulation was conserved in both sexes, as vitamin B6 supplementation fully rescued glucose intolerance in males and females alike.

Accumulating evidence indicates that preconception environmental exposures in fathers, including diet [1,4-9], psychological stress[10], physical exercise[2,11], and gut microbiota dysbiosis [12], can shape the physiological and behavioural phenotypes of offspring via sperm-borne epigenetic mechanisms. In this study, we provide converging evidence that sperm DNA methylation is a critical mediator of metformin's intergenerational effects. We identified 937 Met-DMRs and 2018 HFD-DMRs that were highly correlated with offspring metabolic phenotypes and sensitive to metformin use or cessation. Notably, the vast majority of these Met-DMRs were located in non-imprinted genomic regions, challenging the traditional view that imprinted loci are the primary carriers of paternal intergenerational epigenetic information.

Sperm-derived non-coding RNAs, encompassing microRNAs[1,11,13-16], tRNA-derived small RNAs (tsRNAs) [17,18], mitochondrial RNAs (mt-RNAs)[4], piwi-interacting RNAs (piRNAs)[12], and long non-coding RNAs (lncRNAs)[10], have been identified as pivotal molecular mediators that transmit paternal environmental cues to subsequent generations. Critically, multiple studies by others and our group have demonstrated that the altered ncRNA profiles in sperm resulting from paternal exposures can recapitulate offspring phenotypes when these RNAs are microinjected into zygotes[1,10,14,15,17,19]. We fully acknowledge the importance of sperm ncRNAs in paternal intergenerational inheritance. Comprehensive profiling and functional validation of sperm ncRNAs, including their potential synergistic effects with DNA methylation, will be a major focus of our future studies.

At the metabolic level, our study identifies dysregulation of vitamin B6-dependent kynurenine pathway as a key downstream mechanism linking paternal metformin exposure to offspring insulin resistance. Pyridoxal-5'-phosphate (PLP), the active form of vitamin B6, serves as a cofactor for over 140 mammalian enzymes regulating diverse metabolic processes. We demonstrate that reduced hepatic PLP levels in Met-F1 offspring preferentially impair kynureninase activity, diverting flux toward xanthurenic acid (XA)

synthesis, which directly inhibits muscle insulin signaling and induces glucose intolerance. While vitamin B6 supplementation corrects this cofactor deficiency and fully rescues glucose intolerance, we acknowledge that its beneficial effects may extend beyond the kynurenine pathway, as PLP restoration would normalize all PLP-dependent enzyme activities. This finding nevertheless provides a promising nutritional intervention to mitigate the adverse intergenerational effects of paternal metformin exposure.

Several limitations of this study should be acknowledged. First, all experiments were performed in a rat model, and the translational relevance to humans requires further validation in prospective clinical cohorts. Second, the precise molecular mechanism by which sperm DNA methylation alterations regulate hepatic PLP metabolism in offspring remains to be elucidated. Third, we did not perform functional validation of individual Met-DMRs, which would be required to establish a direct causal link between specific epigenetic marks and offspring phenotypes. Finally, we only assessed the effects of a single dose and duration of metformin treatment; dose-response and long-term exposure studies are needed to fully characterize the intergenerational effects of this drug. But overall, our findings challenge the prevailing assumption of universal preconceptional metformin safety and highlight the need for careful consideration of paternal medication use in reproductive health planning.

Materials and Methods

Animal care and ethics statement

All animal procedures were performed in strict accordance with the Guide for the Care and Use of Laboratory Animals (National Institutes of Health, USA) and approved by the Experimental Animal Ethics Committee of the School of Basic Medical Sciences, Fudan University (20220228-078). Specific-pathogen-free (SPF) Sprague-Dawley (SD) rats were obtained from Shanghai Slack Laboratory Animal Co., Ltd. Animals and their offspring were housed in a barrier-maintained SPF facility under controlled conditions: 4 rats per cage, 12-h light/dark cycle, ambient temperature of 22 ± 2 °C, and relative humidity of 55 ± 10%.

Standard chow (11.4% lipid-derived energy; Cat. #XTC01WC-001, Jiangsu Xietong Pharmaceutical Biotechnology Co., Ltd., China) and sterile water were provided ad libitum unless otherwise specified.

Paternal exposure model and mating protocol

This study employed three independent paternal breeding cohorts (Batch A, B, and C) with distinct experimental objectives, all following identical animal handling and treatment standards (Supplementary Fig. S12a).

Batch A (Core Discovery Cohort)

Designed to systematically evaluate the intergenerational effects of paternal metformin exposure under normal dietary conditions. Ten-week-old male SD rats were maintained on a standard control diet (CND; 11.4% energy from lipids) throughout the study. They were randomly assigned to two groups receiving daily oral gavage (09:00-11:00) for 21 consecutive days: Vehicle (Veh): 1 ml/kg normal saline. Metformin (Met): 200 mg/kg metformin hydrochloride (Cat. #1115-70-4, Aladdin, China) dissolved in saline. The metformin dose was calculated based on body surface area conversion from the human equivalent dose (clinical maintenance dose: 2000-2500 mg/day[20]) and corroborated by prior preclinical studies[21-23].

Batch B (Context and Reversibility Cohort)

Designed to investigate the diet-dependency and reversibility of metformin's intergenerational effects. Four-week-old male SD rats were randomized to either CND or a high-fat diet (HFD; 45% energy from lipids, Cat. #D12451, Research Diets, USA). After 6 weeks of dietary intervention, rats were further divided into six experimental groups: CND-Veh, CND-Met (same treatment as Batch A); HFD-Veh, HFD-Met (HFD-fed rats receiving vehicle or metformin); Veh-WD, Met-WD (CND-fed rats receiving vehicle or metformin, followed by a 3-week drug washout period with no gavage).

Batch C (Mechanistic Validation Cohort)

Dedicated to vitamin B6 intervention studies. Four-week-old male SD rats were maintained on CND and treated with vehicle or metformin as described for Batch A (n=8 per group). This cohort was generated

after the discovery of kynurenine pathway dysregulation in Batch A to validate the therapeutic potential of vitamin B6 supplementation.

Following treatment (or washout) completion, male rats were co-housed with age-matched virgin females at a ratio of 1:2 for 48 hours to allow mating. Males were then removed, and successful mating was confirmed by the presence of a vaginal plug.

Pregnant dams were singly housed, and the date of birth, sex ratio, and birth weight of offspring were recorded. Litter size was standardized to 8 pups per dam on postnatal day 0 (PND 0); Litters with fewer than 8 pups were excluded from the study. Offspring were weaned on PND 28, separated by sex, and housed in groups of 4. The paternal modeling protocol was identical for the same treatment group across different batches; An overview of the animal experimental design for Batches A, B, and C is provided in the schematic figure: Overview of the animal experimental design (Supplementary Fig. S12a). For detailed information on group allocation, individual animal tracking, and the timeline of experiments across F0, F1, and F2 generations, see Supplementary Data 2.xlsx.

Vitamin B6 supplementation in F1 offspring

At 8 weeks of age, F1 offspring from Batch C were randomly divided into two groups and subjected to a 4-week intervention via daily drinking water: the control group received sterile water, while the experimental group was provided with water supplemented with vitamin B6 (2 g/L; Cat. #24102, Henan Kangte Biotechnology Co., Ltd., China) for ad libitum consumption. All solutions were freshly prepared and replaced daily.

Body composition analysis

At 17 weeks of age, body fat and lean mass were quantified using quantitative magnetic resonance (QMR) with a QMR06-90H-PRO instrument (Niumai Analysis, Jiangsu, China).

Glucose and insulin tolerance tests

Rats underwent a 1-week acclimatization period with daily handling prior to testing. For the intraperitoneal glucose tolerance test (IPGTT), animals were fasted for 12 h and injected with 20% glucose (2 g/kg body weight; Cat. #D9434, Sigma-Aldrich, USA). Blood glucose was collected at 0, 15, 30, 60, and 120 min via tail nick and measured using a glucometer (Cat. #GA-3, Sinocare, China). For the insulin tolerance test (ITT), rats were fasted for 6 h and injected intraperitoneally with insulin (0.75 IU/kg; Cat. #HY-P0035, MedChemExpress, USA). Glucose levels were monitored at the same timepoints. Area under the curve (AUC) was calculated for both tests as a composite index of glycemic response.

Histological analyses

At 17 weeks of age, scapular brown adipose tissue (BAT), inguinal white adipose tissue (iWAT), gonadal white adipose tissue (gWAT), and the left lateral liver lobe were dissected, trimmed to ~5 mm^3 blocks, and fixed in 4% paraformaldehyde for 24 h. Adipose tissues were paraffin-embedded, sectioned (5 μm), and stained with hematoxylin and eosin (HE) by Biossci Co., Ltd. Adipocyte area was quantified 200-250 cells per field across three random fields per sample using ImageJ. Liver tissues were embedded in OCT compound, cryosectioned (10 μm; Leica CM3050), and stained with HE or Oil Red O (Cat. #C0105S/C0158S, Beyotime, China). Hepatic steatosis was assessed as the percentage of Oil Red O–positive area per field using Image Pro Plus 6.0.

Serum HOMA indices and lipid profiling

Fasting blood was collected from the tail vein, allowed to clot for 1 h at room temperature, and centrifuged (1,500 × g, 15 min, 4 °C). The separated serum was stored at −80 °C until analysis. Fasting insulin was measured using a rat insulin ELISA kit (Cat. #KE20008, Proteintech, USA), and fasting glucose was determined with a glucometer. Homeostatic model assessment of insulin resistance (HOMA-IR) was calculated as follows: HOMA-IR = fasting glucose (mmol/L) × fasting insulin (mU/L) / 22.5.

Serum levels of triglycerides (TG), high-density lipoprotein cholesterol (HDL-C), and low-density lipoprotein cholesterol (LDL-C) were measured in duplicate using commercial assay kits (TG: Cat. #A110-

1-1; HDL-C: Cat. #A112-1-1; LDL-C: Cat. #A113-1-1, Nanjing Jiancheng Bioengineering Institute, China).

Metabolic cage studies

Energy metabolism of mice was monitored using the OxyletPro system (Panlab, Harvard Apparatus). Following a 24h acclimatization period, data were recorded over the subsequent 48 h for analysis. Parameters including food intake, locomotor activity, and the respiratory quotient (RQ; calculated as VCO_2/VO_2) were measured. Environmental conditions matched those of the housing facility.

Western blot analysis

At 17 weeks, F1 rats received an intraperitoneal injection of insulin (1.5 U/kg) or saline. 10 minutes later, liver and gastrocnemius muscles were harvested. Tissues were homogenized in ice-cold RIPA buffer (Cat. #P0013B, Beyotime, China) containing protease and phosphatase inhibitors. Protein concentration was determined by BCA assay (Cat. #23227, Thermo Fisher). Equal amounts of protein (20 μg) were separated on 4-20% Tris-Glycine gels (Cat. #P0468S/P0469S, Beyotime, China) and transferred to PVDF membranes (Cat. #FFP32, Beyotime, China). Membranes were blocked with 5% BSA and probed overnight at 4 °C with primary antibodies: β-Actin (1:5000, Cat. #AC026, ABclonal, China), total Akt (1:1000, Cat. #4685, Cell Signaling Technology, USA), and phospho-Akt (Ser473, 1:2000, Cat. #4060S, Cell Signaling Technology, USA). Fluorescent secondary antibodies (DyLight 680: Cat. #611-144-002; DyLight 800: Cat. #611-145-002, Rockland Immunochemicals, USA) were used for detection on an Odyssey CLx system (LI-COR, USA). Band intensities were quantified using Image Studio V3.1 and normalized to β-Actin.

Sperm DNA RRBS library preparation and analysis

Sperm were isolated from cauda epididymis and vas deferens, purified through somatic cell lysis (0.1% SDS, 0.5% Triton X-100), and stored at –80 °C until use. Reduced-representation bisulfite sequencing (RRBS) libraries were constructed by E-Gene Co., Ltd. Briefly, 1 μg genomic DNA (spiked with

unmethylated lambda DNA) was digested with MspI (Cat. #R0106M, New England Biolabs, USA) at 37 °C for 16 h. DNA was end-repaired, A-tailed, and ligated to methylated adaptors. Bisulfite conversion was performed using the EZ DNA Methylation-Gold Kit (Cat. #D5005, Zymo Research, USA).The converted DNA was then amplified by PCR in a 50 µL reaction containing 20 µL adapter-ligated DNA, 1 µL 10 mM dNTPs, 5 µL 10× buffer, 0.8 µL JumpStart™ Taq DNA Polymerase, 2 µL PCR primers, and 21.2 µL water, under the following cycling conditions: 94 °C for 1 min; 12 cycles of 94 °C for 30 s, 58 °C for 30 s, 72 °C for 40 s; and a final extension at 72 °C for 5 min. Libraries were size-selected and quantified using a Bioanalyzer system (Agilent) and real-time PCR, respectively, before sequencing on an Illumina platform. Sequencing reads were aligned to the UCSC rat reference genome (rn6). Methylation levels (beta-values) were calculated as C/(C+T) using BSMAP[24]. Differentially methylated regions (DMRs) were identified with MethylKit and metilene[25], respectively ($P < 0.05$).

RNA sequencing

Total RNA was extracted from gastrocnemius using TRIzol (Cat. #15596026, Thermo Fisher Scientific, USA), with rRNA depleted with Ribo-off rRNA Depletion Kit (Cat. #NR406, Vazyme, China). Libraries were prepared with the Total RNA Library Prep Kit (Cat. #NR606, Vazyme, China) and sequenced by Genewiz on an Illumina HiSeq 3000. Reads were trimmed (Trimmomatic V0.32), aligned to rn6.0 (HISAT2 V2.0.2), and quantified (featureCounts V1.6.2). Differential expression was analyzed with DESeq2 (V1.46.0)[26] ($|log_2FC| > 0.6$, $Pvalue < 0.05$). KEGG enrichment was performed with clusterProfiler (4.14.6)[27].

Publicly available dataset processing and analysis

PPARγ target genes were retrieved from KnockTF 2.0 database[28]. Gene set enrichment analysis (GSEA) was then performed to evaluate the enrichment of these PPARγ target genes within the transcriptional profiles of offspring muscle tissues.

Human plasma metabolomics data were obtained from a published cohort study[29] (Teng Huang et al., 2022) that included adults with a wide range of BMI. We performed a post hoc reanalysis of publicly available raw plasma metabolomics data, focusing on circulating pyridoxal-5'-phosphate (PLP) and xanthurenic acid (XA), together with BMI. Individuals were categorized into lean (BMI 18-24 kg/m^2), overweight (24-28 kg/m^2), and obese (⩾28 kg/m^2). Subjects with missing metabolite or BMI data, or extreme outliers (±3 SD) were excluded. Associations between PLP/XA and BMI were evaluated using Pearson correlation and linear regression, adjusted for age and sex. All analyses were performed using R, with a two-sided $P < 0.05$ considered significant.

Ethics approval

The human plasma analysis uses only publicly available, fully anonymized data and does not constitute human subjects research requiring additional ethics approval. The original study[29] obtained informed consent from all participants, and all procedures involving human subjects were conducted in accordance with the guidelines of the National Institutes of Health (NIH) and approved by the Institutional Review Board (IRB) of Tongji Hospital (Approval Nos. TJ-IRB20160601 and TJ-IRB20160602).

Untargeted metabolomics

Plasma and liver samples were collected from 17-week-old F1 rats (Batch A) at 9:00 AM and stored at −80 °C until analysis. Untargeted metabolomic profiling was performed by Novogene Technology Co., Ltd. Briefly, samples were extracted in 80% methanol, centrifuged, and analyzed on a high-resolution mass spectrometer. Metabolites with CV < 30% in QC samples were retained.

Differential metabolites were identified based on the criteria of variable importance in projection (VIP) > 1.0 and a P-value < 0.05. These differential metabolites were further analyzed using MetaboAnalyst 6.0 (https://www.metaboanalyst.ca) for pathway enrichment.

Targeted metabolomics

Plasma samples were collected from 12-week-old F1 rats (Batch C) at 9:00 AM and stored at −80 °C until analysis. Targeted metabolomic analysis of tryptophan metabolites was conducted by MetWare Biotechnology Co., Ltd. For sample pretreatment, 50 μL of sample was mixed with 250 μL of methanol containing 10 μL of internal standard working solution (250 ng/mL), vortexed for 3 min, incubated at −20 °C for 30 min, and centrifuged (12,000 rpm, 10 min, 4 °C). A 100 μL aliquot of supernatant was used for LC-tandem MS (LC-MS/MS). Metabolites were identified by matching against the MetWare Database (MWDB), which was established using authentic standards. Quantification was performed with MultiQuant 3.0.3 software by integrating chromatographic peaks and calculating absolute concentrations from external standard curves.

Xanthurenic acid (XA) functional validation

In vitro: Gastrocnemius muscles were dissected from 4-week-old male Sprague Dawley (SD) rats and minced under sterile conditions. Primary myoblast isolation and culture followed a published standardized protocol[30]. Briefly, muscle tissues were digested and cultured in DMEM/F12 (Cat. #11320033, Gibco, USA) with 15% FBS (Cat. #WS500T, Ausbian, Australia) and 1% penicillin/streptomycin (Cat. #B541011, Sangon Biotech, China). Myoblasts were purified by differential adhesion and subsequently differentiated in DMEM/F12 + 2% horse serum (Cat. #16050130, Thermo Fisher Scientific, USA) + 1% penicillin/streptomycin. Differentiated myotubes were used for subsequent assays. Cells were serum-starved, treated with XA (0, 10, 30, 100 nM; Cat. #HY-W014666, MedChemExpress, USA) for 30 min, stimulated with insulin (30 nM) for 15 min, and lysed for Western blot.
In vivo: Twelve-week-old SD rats received intraperitoneal XA (100 mg/kg in saline + 1 mM ascorbate, pH 7.4) or vehicle. IPGTT was performed 1 h post-injection.

Statistical analysis

Data are presented as mean ± SEM. Analyses were performed in GraphPad Prism (v9.0) and R (v4.3.3). Group comparisons used one- or two-way ANOVA with Bonferroni's *post hoc* test; two-group comparisons

used two-tailed Student's *t*-test. For F1/F2 offspring, litter effects were evaluated using intraclass correlation coefficients (ICC), and linear mixed-effects models included litter as a random effect. $P < 0.05$ was considered significant.

Bivariate Phenotypic Analysis: To integrate body weight and glucose tolerance into a unified metabolic framework, we calculated Z-scores for body weight and IPGTT AUC for each individual relative to the control group (CND-Veh). These standardized scores were plotted bi-dimensionally. Geometric centroids were calculated for each experimental group to visualize the shift in metabolic set-points, and the Euclidean distance between centroids was used to quantify the magnitude of phenotypic displacement.

Extended Data Figures

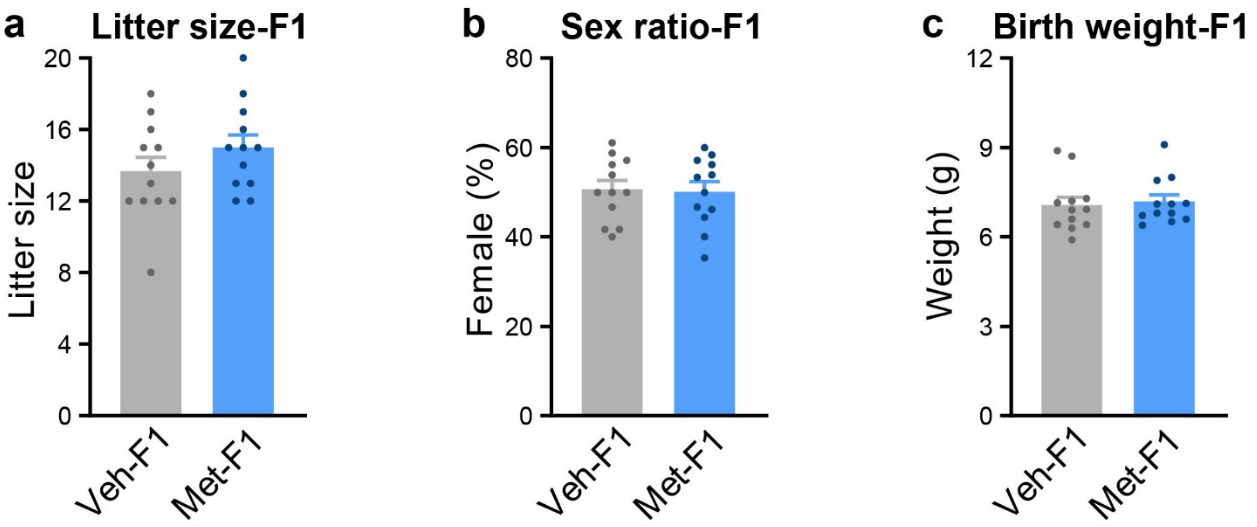

Supplementary Figure 1. Effects of paternal metformin on birth parameters in F1 offspring. **(a-c)** Birth parameters: (**a**) litter size, (**b**) sex ratio (female/total), and (**c**) body weight at postnatal day 0 (P0) (n = 12 per group). Data are presented as mean ± SEM. Significance thresholds: $P < 0.05$ (*), $P < 0.01$ (**), $P < 0.001$ (***).

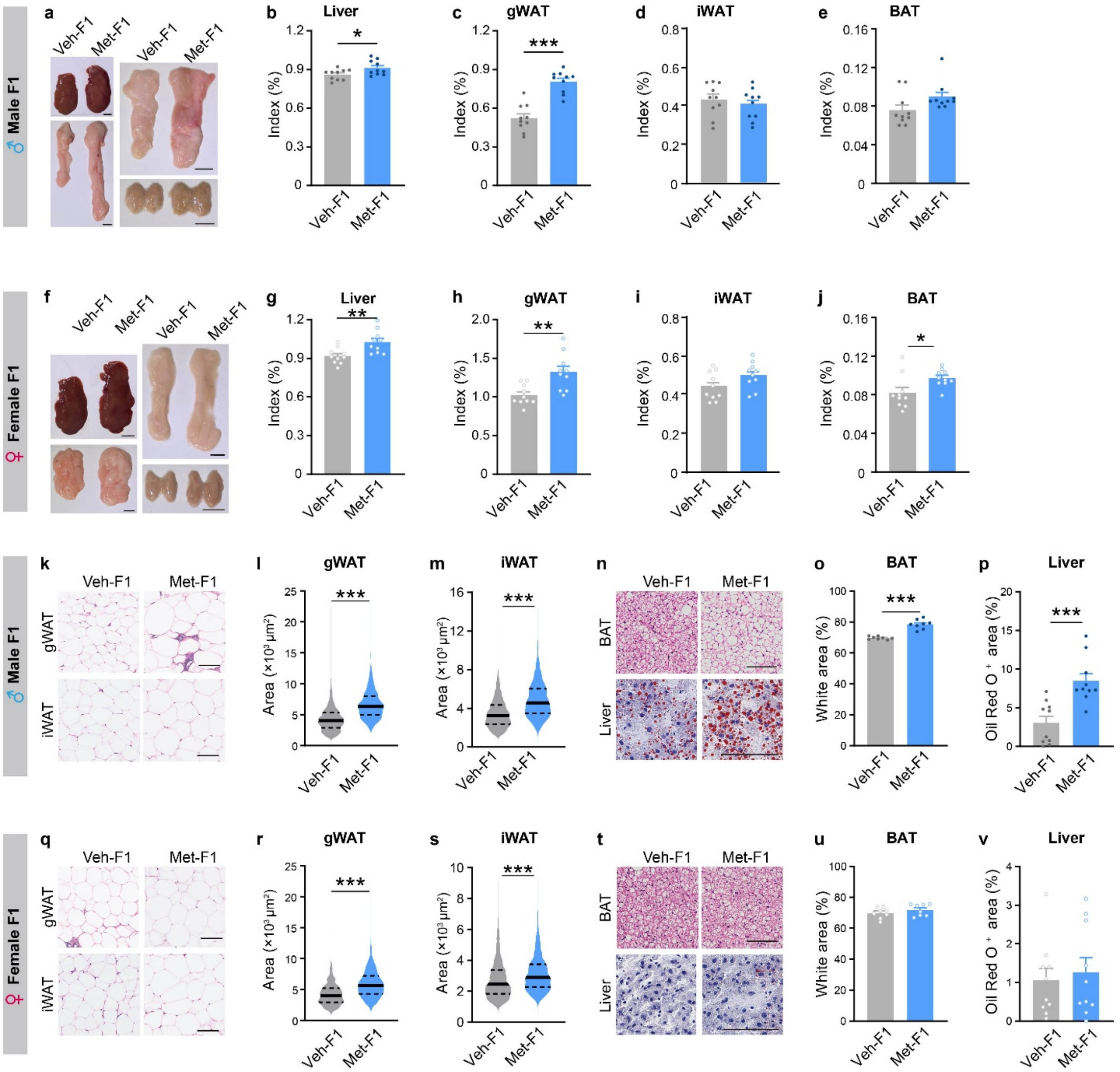

Supplementary Figure 2. Comprehensive characterization of adipose tissue, liver morphology in F1 offspring.

(a-j) Representative morphology and organ index of the liver, gonadal white adipose tissue (gWAT), inguinal white adipose tissue (iWAT), and brown adipose tissue (BAT) in F1 male **(a-e)** and female **(f-j)** offspring (Veh-F1, n = 10 from 10 litters, Met-F1, n = 10 from 10 litters; scale bar, 1 cm). **(k–v)** Histological staining and related indicators of adipose tissue and liver in F1 male **(k–p)** and female **(q–v)** offspring: **(k, q)** Representative H&E-stained sections of gWAT and iWAT (scale bar, 100 μm); **(l, r)** adipocyte cross-sectional area of gWAT; **(m, s)** adipocyte cross-sectional area of iWAT; **(n, t)** Representative H&E-stained

BAT sections and Oil Red O-stained liver sections (scale bar, 100 μm); **(o, u)** white vacuole area in BAT; **(p, v)** hepatic Oil Red O-positive area. (adipose tissue: Veh-F1, $n = 8$ from 8 litters, Met-F1, $n = 8$ from 8 litters; liver: Veh-F1, $n = 10$ from 10 litters, Met-F1, $n = 10$ from 10 litters). Data are presented as mean ± SEM. Significance thresholds: $P < 0.05$ (*), $P < 0.01$ (**), $P < 0.001$ (***).

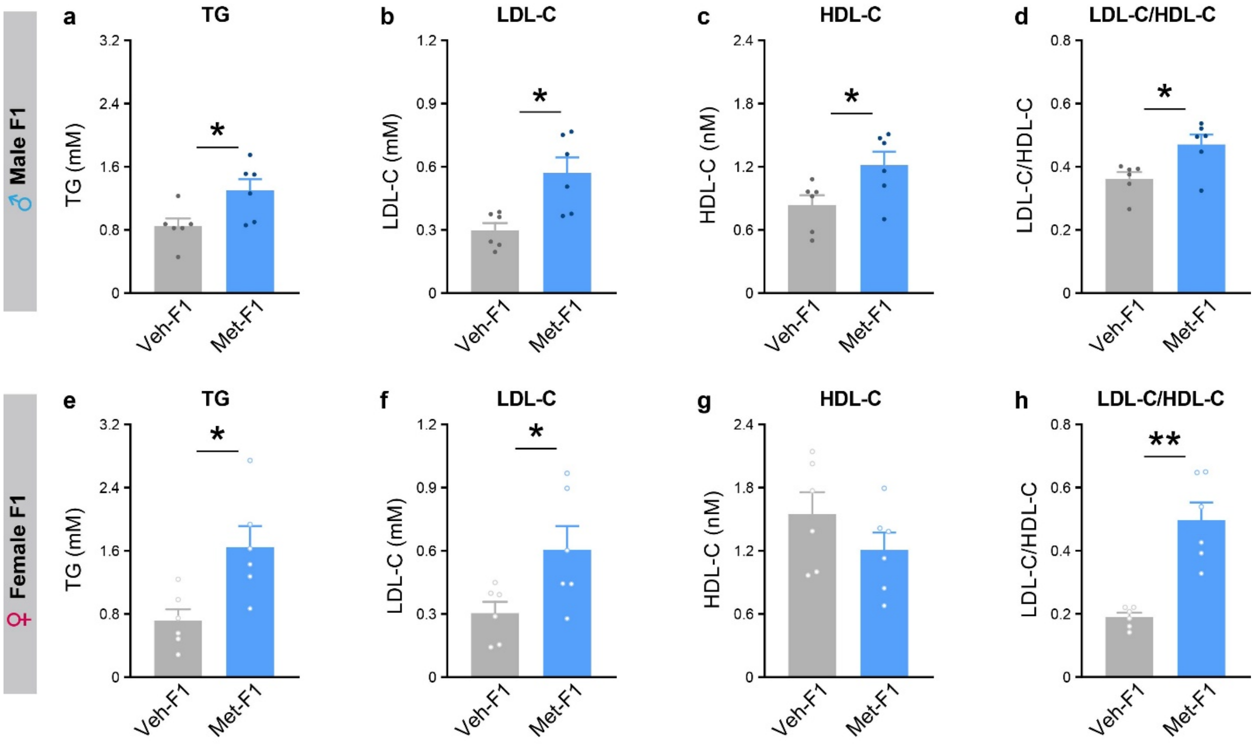

Supplementary Figure 3. Serum lipid profiles in F1 offspring.

(a-h) Serum lipid profiles in F1 male **(a-d)** and female **(e-h)** offspring: **(a, e)** Triglycerides (TG); **(b, f)** low-density lipoprotein cholesterol (LDL-C); **(c, g)** high-density lipoprotein cholesterol (HDL-C); **(d, h)** LDL-C/HDL-C ratio. (Veh-F1, $n = 6$ from 6 litters, Met-F1, $n = 6$ from 6 litters). Data are presented as mean ± SEM. Significance thresholds: $P < 0.05$ (*), $P < 0.01$ (**), $P < 0.001$ (***).

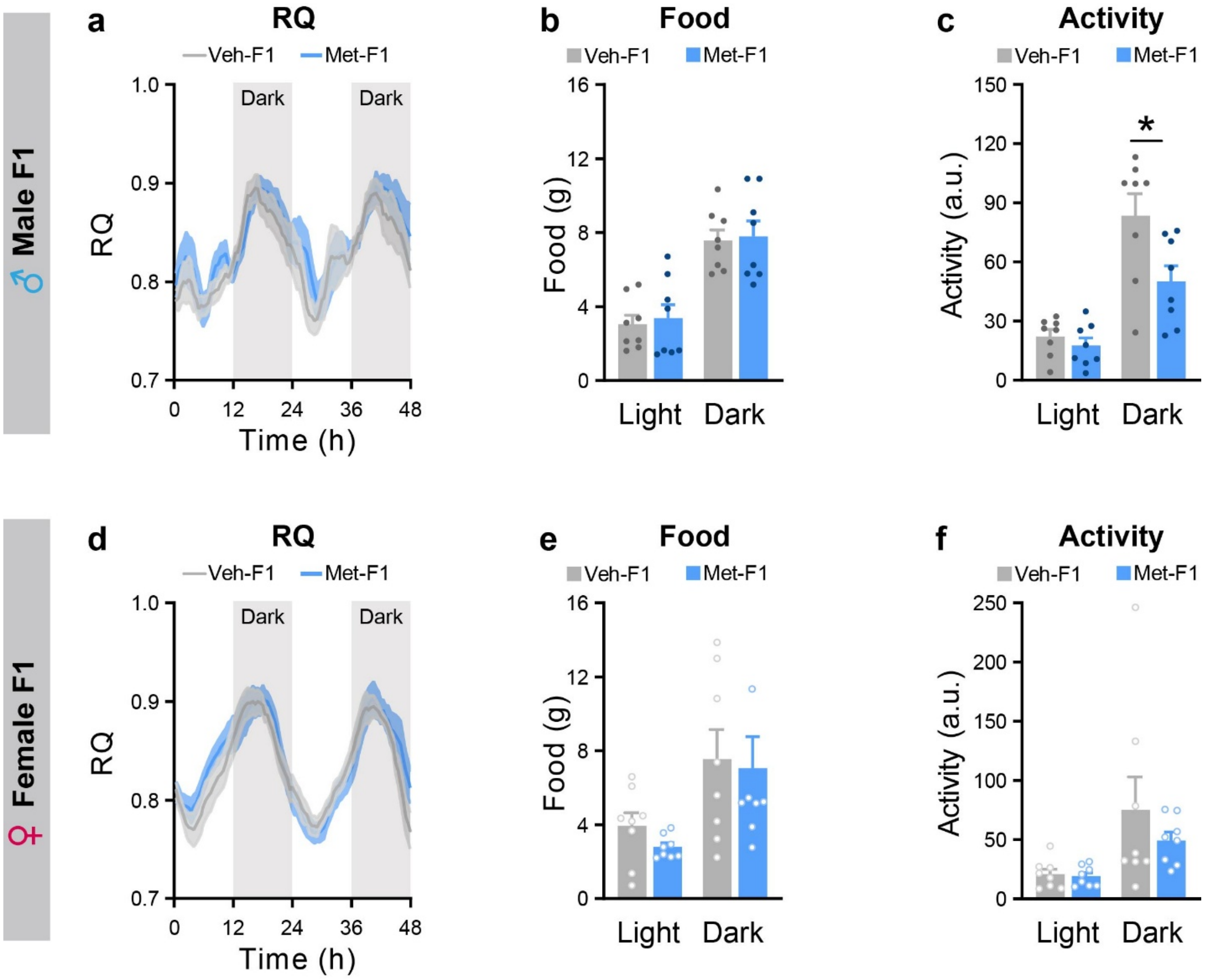

Supplementary Figure 4. Metabolic phenotyping and energy expenditure profile in F1 offspring. (**a-f**) Metabolic cage data for F1 males (**a-c**) and females (**d-f**): Respiratory quotient (RQ; **a, d**), cumulative food intake (**b, e**), and locomotor activity (**c, f**) (Veh-F1, *n* = 8 from 8 litters, Met-F1, *n* = 8 from 8 litters). Data are presented as mean ± SEM. Significance thresholds: P < 0.05 (*), P < 0.01 (**), P < 0.001 (***).

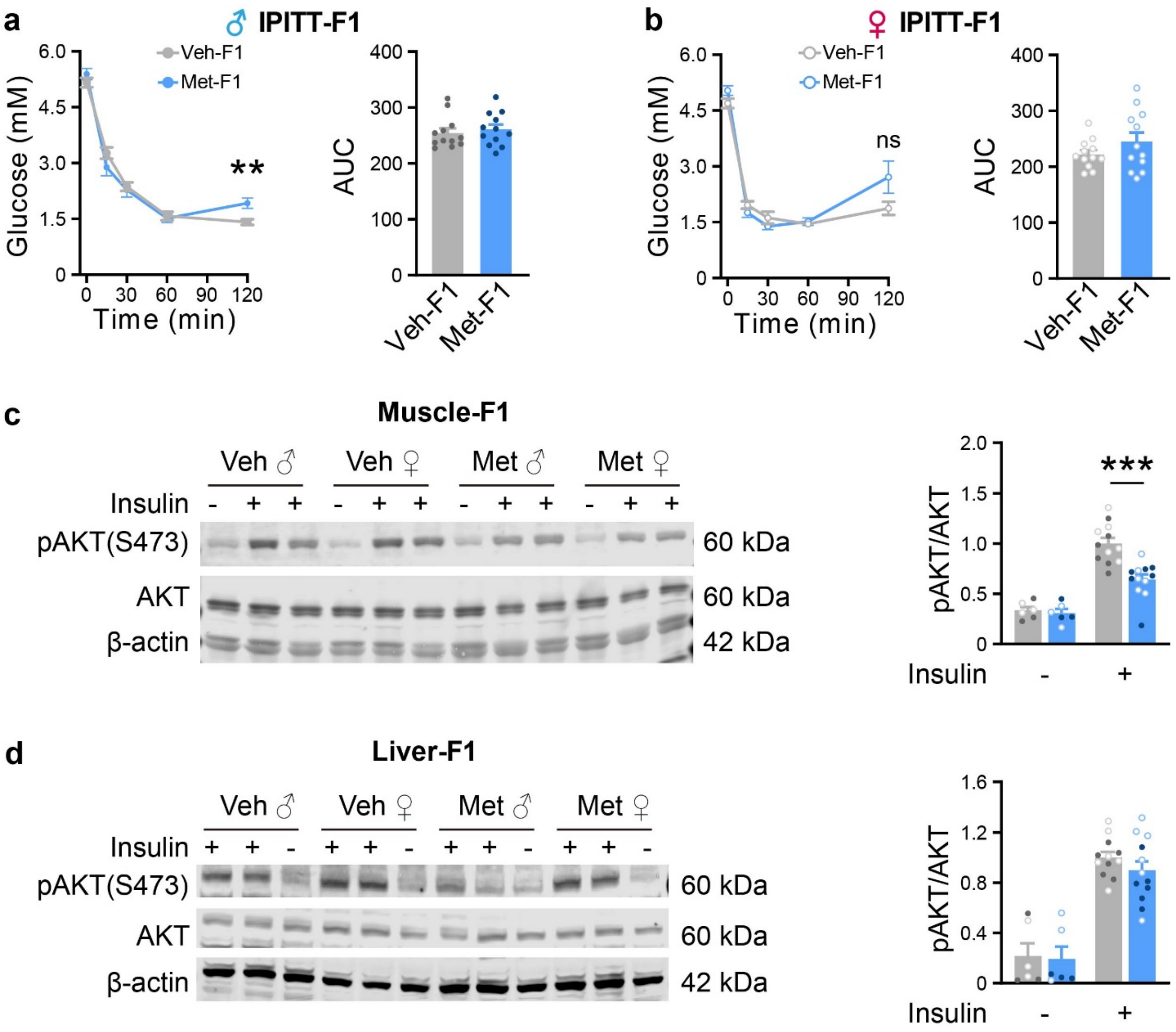

Supplementary Figure 5. Assessment of insulin tolerance and tissue-specific insulin signaling in F1 offspring.

(**a, b**) Intraperitoneal insulin tolerance test (IPITT) curves and corresponding AUC in F1 males (**a**) and females (**b**) (Veh-F1, $n = 12$ from 12 litters, Met-F1, $n = 12$ from 12 litters). (**c, d**) Insulin-AKT signaling analysis in muscle (**c**) and liver (**d**) of F1 offspring. (left) representative immunoblots of phosphorylated AKT (pAKT), total AKT, and β-actin under basal (-) and insulin-stimulated (+) conditions. (right) quantification of the pAKT/AKT ratio (n: insulin$^-$, Veh-F1, $n = 6$ from 6 litters, Met-F1, $n = 6$ from 6 litters; insulin$^+$: Veh-F1, $n = 12$ from 12 litters, Met-F1, $n = 12$ from 12 litters). Data are presented as mean ± SEM. Significance thresholds: $P < 0.05$ (*), $P < 0.01$ (**), $P < 0.001$ (***).

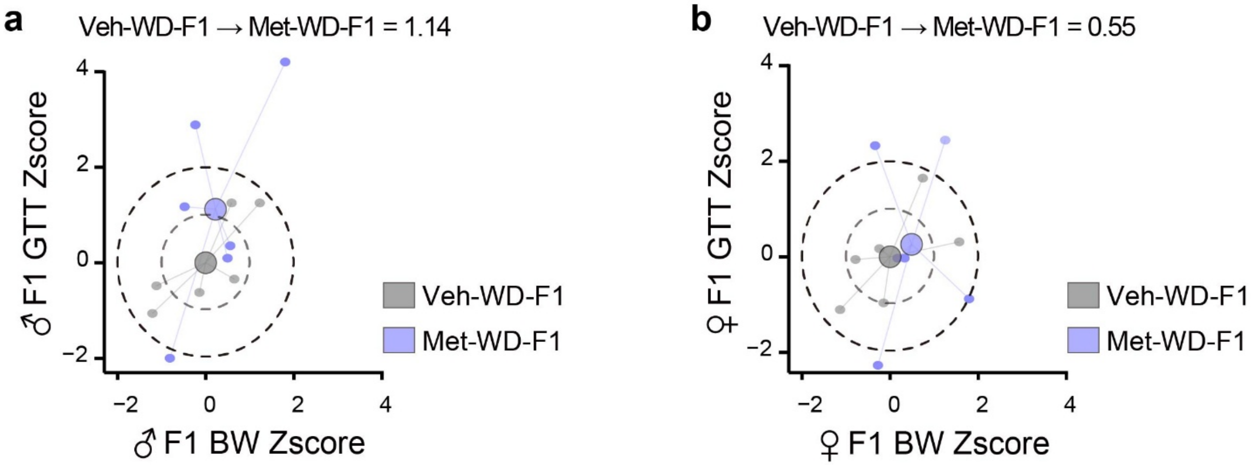

Supplementary Figure 6. Reversibility of paternal metformin-induced metabolic phenotypes in F1 offspring following a washout period.

(**a**, **b**) Bivariate phenotypic analysis of F1 male (**a**) and female (**b**) offspring from the washout cohort, plotted by Z-scores for GTT AUC and body weight. Each small point represents an individual litter, color-coded by paternal treatment group. The geometric centroid for each group is shown as a larger filled symbol (Data are integrated from Figs. 1f, 1g). Data are presented as mean ± SEM. Significance thresholds: $P < 0.05$ (*), $P < 0.01$ (**), $P < 0.001$ (***).

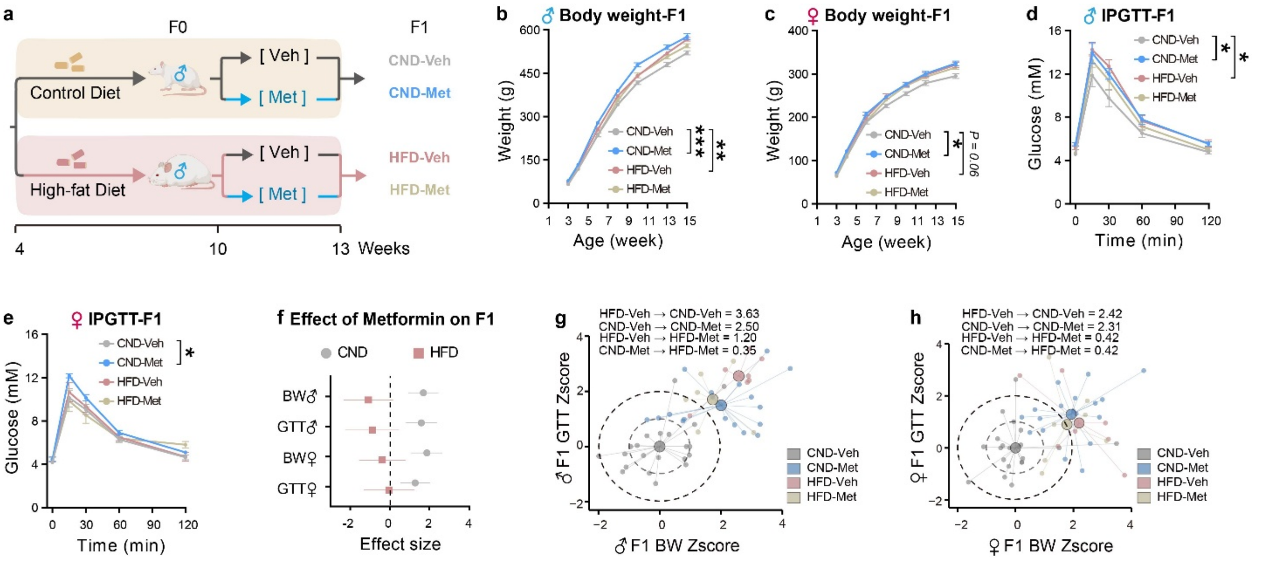

Supplementary Figure 7. Paternal metformin exerts diet-dependent effects on F1 offspring metabolic phenotypes.

(a) Schematic of experimental design: F0 males received vehicle (Veh) or metformin (Met) while maintained on either a control diet (CND) or high-fat diet (HFD), followed by mating to generate F1 offspring. (**b**, **c**) Body weight of F1 male (**b**) and female (**c**) offspring (CND-Veh, n = 18 from 9 litters; CND-Met, n =12 from 6 litters; HFD-Veh, n = 14 from 7 litters; HFD-Met, n = 14 from 7 litters). **(d, e)** IPGTT curves for F1 male (**d**) and female (**e**) offspring (n=6 per group, 6 litters). (**f**) Cohen's *d* effect sizes quantifying the impact of paternal metformin on key metabolic traits in F1 offspring (BW, body weight; GTT, AUC of GTT). (**g, h**) Bivariate phenotypic analysis of F1 male (**g**) and female (**h**) offspring across experimental groups, plotted by Z-scores for GTT AUC and body weight. Each small point represents an individual litter, color-coded by paternal treatment group. The geometric centroid for each group is shown as a larger filled symbol (Data are integrated from Figs. 1b, 1d and Supplementary Figs. 7b-e). Data are presented as mean ± SEM. Significance thresholds: P < 0.05 (*), P < 0.01 (**), P < 0.001 (***).

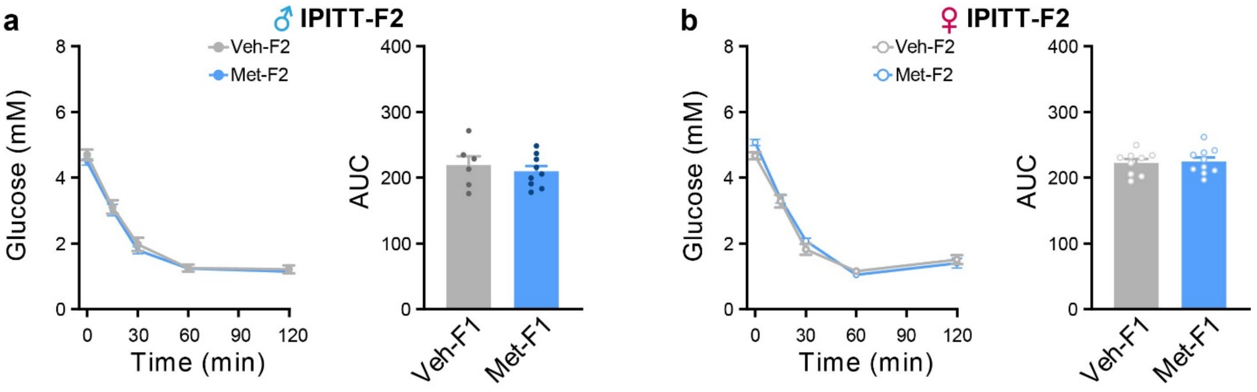

Supplementary Figure 8. Assessment of insulin tolerance in F2 offspring.

(**a, b**) IPITT curves and corresponding AUC in F2 males (**a**) and females (**b**) (males: Veh-F2 $n = 10$ from 10 litters, Met-F2 $n = 11$ from 11 litters; females: Veh-F2 $n = 7$ from 7 litters, Met-F2 $n = 10$ from 10 litters). Data are presented as mean ± SEM. Significance thresholds: $P < 0.05$ (*), $P < 0.01$ (**), $P < 0.001$ (***).

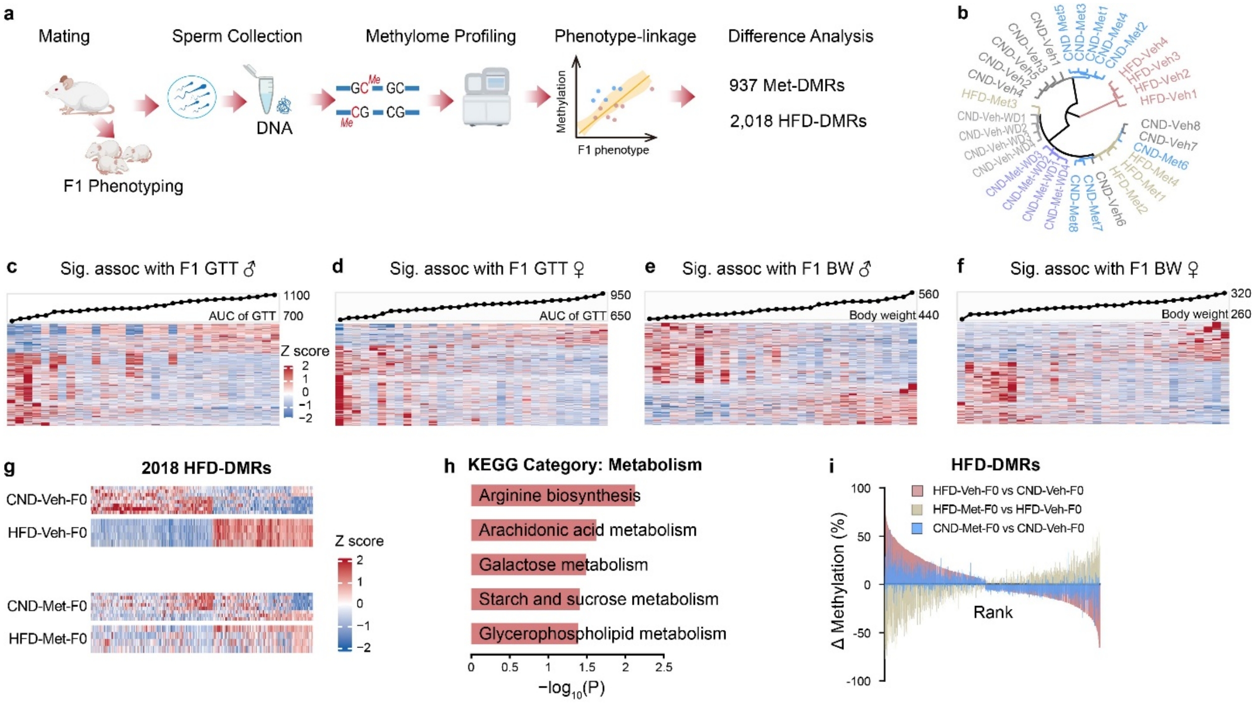

Supplementary Figure 9. Paternal metformin reprograms sperm DNA methylome in a diet- and withdrawal-dependent manner and associates with metabolic alterations in F1 offspring.

(**a**) Schematic of reduced representation bisulfite sequencing (RRBS) workflow applied to sperm from F0 male rats and integrative strategy to identify candidate differentially methylated regions (DMRs) by correlating F0 sperm methylomes with F1 metabolic phenotypes (CND-Veh = 8, CND-Met = 8, HFD-Veh = 4, HFD-Met = 4, CND-Veh-WD = 4, CND-Met-WD = 4). (**b**) Hierarchical clustering of F0 sperm DNA methylation profiles. (**c–f**) Heatmaps display MRs significantly correlated ($P < 0.05$) with GTT AUC or BW in F1 offspring, with samples ordered left to right by increasing phenotype value: (**c**) GTT AUC in males; (**d**) GTT AUC in females; (**e**) BW in males; (**f**) BW in females. **(g)** Heatmap of 2,018 DMRs significantly linked to paternal high-fat diet exposure. (**h**) KEGG pathway enrichment analysis of genes overlapping HFD-associated DMRs. (**i**) Ranking plot of methylation changes of HFD-associated DMRs across three comparisons: HFD-Veh vs. CND-Veh (HFD effect), HFD-Met vs. HFD-Veh (metformin effect under HFD), and CND-Met vs. CND-Veh (metformin effect under CDN).

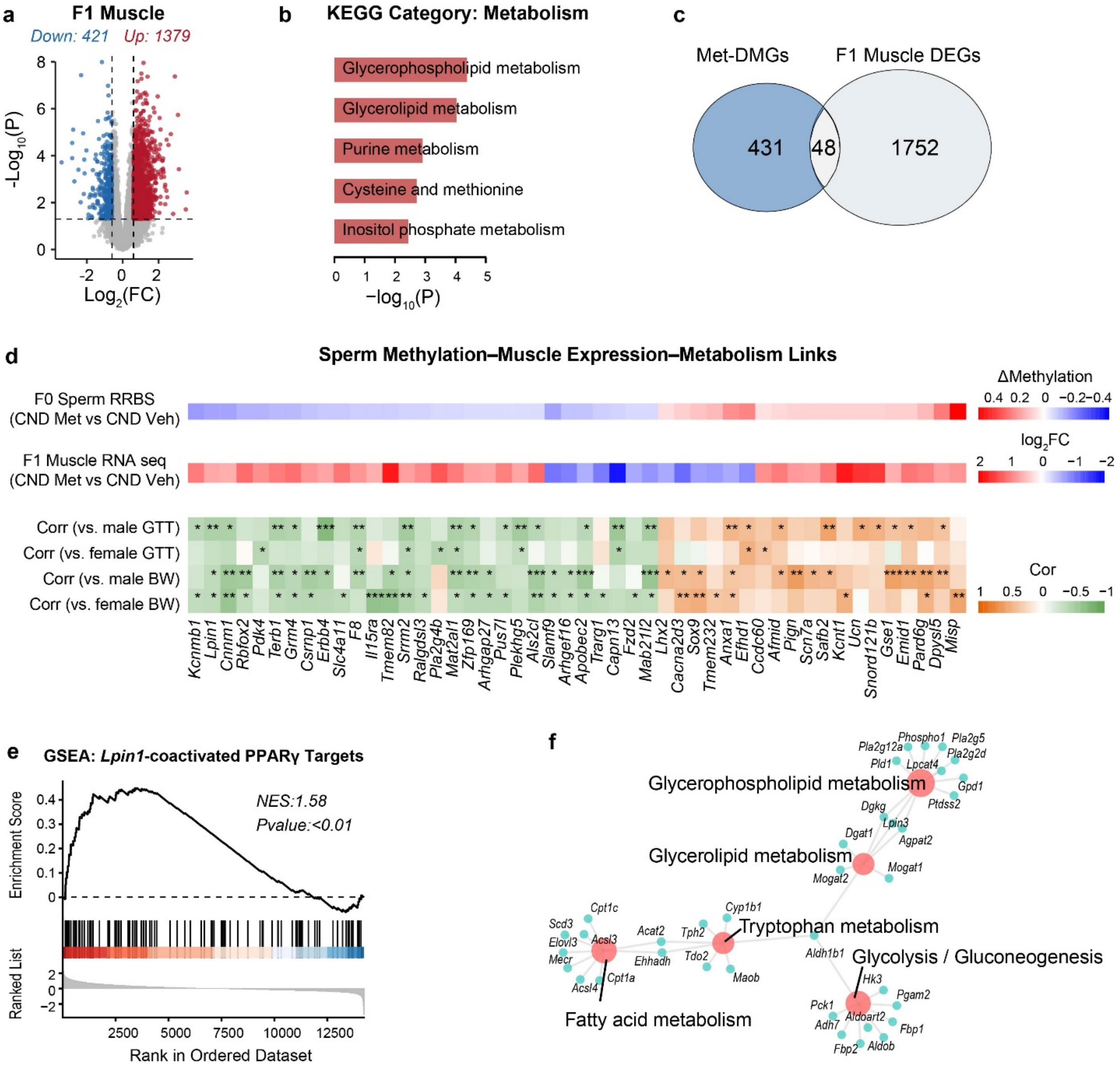

Supplementary Figure 10. Concordant alterations in F0 sperm methylation and F1 muscle PPARG pathway activity following metformin treatment.

(**a**) Volcano plots of differentially expressed genes (DEGs) in F1 muscle (males: Veh-F1, n = 5 from 5 litters, Met-F1, n = 5 from 5 litters; females: Veh-F1, n = 5 from 5 litters, Met-F1, n = 5 from 5 litters). Genes with |log₂ fold change| > 0.6 and P < 0.05 are highlighted in red (upregulated) or blue (downregulated). (**b**) KEGG pathway enrichment analysis results of DEGs in F1 muscle. Top 5 enriched pathways related to metabolic are shown. (**c**) Venn diagram showing overlap between genes associated with metformin-induced

DMRs in F0 sperm and DEGs in F1 muscle. (**d**) Heatmap depicting the overlap of genes identified in panel c, illustrating the relationship between DNA methylation changes in F0 sperm and mRNA expression changes in F1 muscle, as well as the correlation between methylation levels and offspring metabolic traits (Pearson's r; significance: $^*P < 0.05$, $^{**}P < 0.01$, $^{***}P < 0.001$). (**e**) Gene Set Enrichment Analysis (GSEA) of the top 100 PPARG target genes in F1 muscle transcriptomes. NES: Normalized enrichment score. (**f**) Lpin1 functions as a coactivator of PPARG, driving the expression of downstream target genes involved in key metabolic pathways.

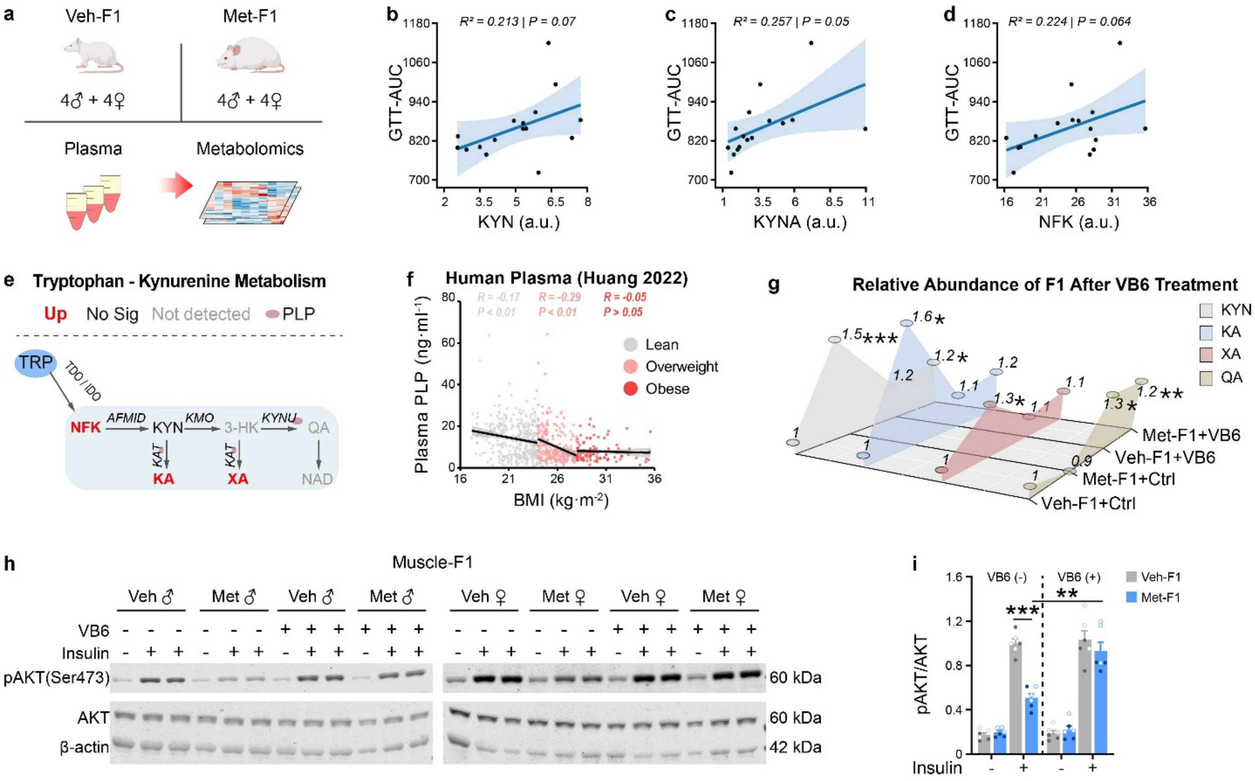

Supplementary Figure 11. Vitamin B6 rescuing kynurenine pathway dysregulation and restoring insulin sensitivity in F1 offspring of metformin‑exposed fathers.

(**a**) Study design and plasma metabolomics workflow: Untargeted LC-MS metabolomic profiling was performed on plasma from 17-week-old F1 offspring (males: Veh-F1, *n* = 4 from 4 litters, Met-F1, *n* = 4 from 4 litters; females: Veh-F1, *n* = 4 from 4 litters, Met-F1, *n* = 4 from 4 litters). (**b-d**) Linear regression analyses between plasma concentrations of kynurenine pathway metabolites and glucose tolerance (GTT AUC) in F1 offspring: (**b**) KYN; (**c**) KYNA; (**d**) NFK. (**e**) Schematic diagram of altered tryptophan-kynurenine metabolism in F1 offspring. (**f**) Correlation analysis between plasma pyridoxal-5-phosphate (PLP) levels and body mass index (BMI) in human plasma. (lean: BMI 18-24, *n* = 354; overweight: BMI 24-28, *n* = 268; obese: BMI ≥28, *n* = 113) (data from Teng Huang et al., 2022). (**g**) Relative abundance of tryptophan–kynurenine (Trp–KYN) pathway metabolites in F1 plasma after VB6 treatment (Veh-F1+Ctrl, *n* = 8 from 8 litters, Met-F1+Ctrl, *n* = 8 from 8 litters, Veh-F1+VB6, *n* = 8 from 8 litters, Met-F1+VB6, *n* = 8 from 8 litters; statistically compared to the Veh-F1+Ctrl group). (**h, i**) Insulin-AKT signaling analysis

in F1 muscle afterVB6 treatment. (**h**) Representative Western blots for phosphorylated AKT (Ser473), total AKT, and β-actin under basal (–) and insulin-stimulated (+) conditions. (**i**) Quantification of pAKT/AKT ratio (*n*: insulin–/insulin+, Veh-F1+Ctrl, $n = 6$ from 6 litters, Met-F1+Ctrl, $n = 6$ from 6 litters, Veh-F1+VB6, $n = 6$ from 6 litters, Met-F1+VB6, $n = 6$ from 6 litters). Data are presented as mean ± SEM. Significance thresholds: $P < 0.05$ (*), $P < 0.01$ (**), $P < 0.001$ (***).

a

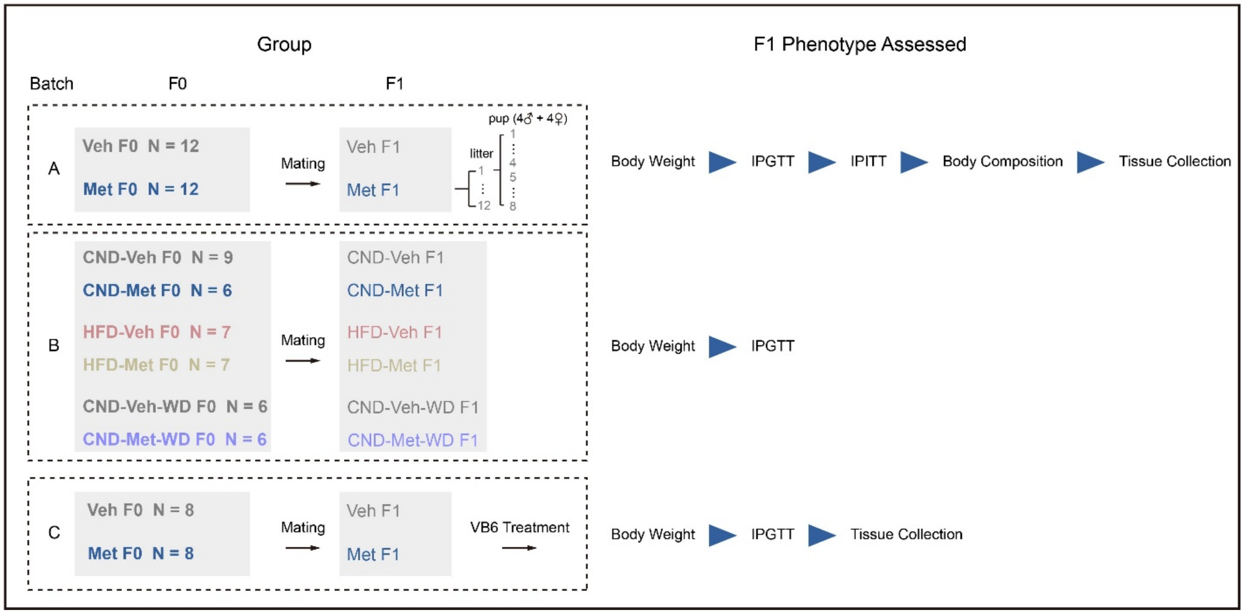

Supplementary Figure 12. Overview of the animal experimental design.

(**a**) An overview of the animal experimental design for Batches A, B, and C.

References

1 Huang, C. *et al.* Male obesity causes adipose mitochondrial dysfunction in F(1) mouse progeny via a let-7-DICER axis. *Nature communications* **17**, doi:10.1038/s41467-026-69686-5 (2026).

2 Murashov, A. K. *et al.* Paternal long-term exercise programs offspring for low energy expenditure and increased risk for obesity in mice. *FASEB journal : official publication of the Federation of American Societies for Experimental Biology* **30**, 775-784, doi:10.1096/fj.15-274274 (2016).

3 Liu, W. *et al.* Asymmetric reprogramming capacity of parental pronuclei in mouse zygotes. *Cell reports* **6**, 1008-1016, doi:10.1016/j.celrep.2014.02.018 (2014).

4 Tomar, A. *et al.* Epigenetic inheritance of diet-induced and sperm-borne mitochondrial RNAs. *Nature* **630**, 720-727, doi:10.1038/s41586-024-07472-3 (2024).

5 Bodden, C. *et al.* Intergenerational effects of a paternal Western diet during adolescence on offspring gut microbiota, stress reactivity, and social behavior. *FASEB journal : official publication of the Federation of American Societies for Experimental Biology* **36**, e21981, doi:10.1096/fj.202100920RR (2022).

6 Fontelles, C. C. *et al.* Systemic alterations play a dominant role in epigenetic predisposition to breast cancer in offspring of obese fathers and is transmitted to a second generation. *Scientific reports* **11**, 7317, doi:10.1038/s41598-021-86548-w (2021).

7 Fontelles, C. C. *et al.* Paternal overweight is associated with increased breast cancer risk in daughters in a mouse model. *Scientific reports* **6**, 28602, doi:10.1038/srep28602 (2016).

8 McPherson, N. O. *et al.* Paternal under-nutrition programs metabolic syndrome in offspring which can be reversed by antioxidant/vitamin food fortification in fathers. *Scientific reports* **6**, 27010, doi:10.1038/srep27010 (2016).

9 Ryan, D. P. *et al.* A paternal methyl donor-rich diet altered cognitive and neural functions in offspring mice. *Molecular psychiatry* **23**, 1345-1355, doi:10.1038/mp.2017.53 (2018).

10 Hoffmann, L. B. *et al.* Chronically high stress hormone levels dysregulate sperm long noncoding RNAs and their embryonic microinjection alters development and affective behaviours. *Molecular psychiatry* **29**, 590-601, doi:10.1038/s41380-023-02350-2 (2024).

11 Short, A. K. *et al.* Exercise alters mouse sperm small noncoding RNAs and induces a transgenerational modification of male offspring conditioned fear and anxiety. *Translational psychiatry* **7**, e1114, doi:10.1038/tp.2017.82 (2017).

12 Masson, B. A. *et al.* Depletion of the paternal gut microbiome alters sperm small RNAs and impacts offspring physiology and behavior in mice. *Brain, behavior, and immunity* **123**, 290-305, doi:10.1016/j.bbi.2024.09.020 (2025).

13 Fullston, T., Ohlsson-Teague, E. M., Print, C. G., Sandeman, L. Y. & Lane, M. Sperm microRNA Content Is Altered in a Mouse Model of Male Obesity, but the Same Suite of microRNAs Are Not Altered in Offspring's Sperm. *PloS one* **11**, e0166076, doi:10.1371/journal.pone.0166076 (2016).

14 Grandjean, V. *et al.* RNA-mediated paternal heredity of diet-induced obesity and metabolic disorders. *Scientific reports* **5**, 18193, doi:10.1038/srep18193 (2015).

15 Rodgers, A. B., Morgan, C. P., Leu, N. A. & Bale, T. L. Transgenerational epigenetic programming via sperm microRNA recapitulates effects of paternal stress. *Proceedings of the National Academy of Sciences of the United States of America* **112**, 13699-13704, doi:10.1073/pnas.1508347112 (2015).

16 Short, A. K. *et al.* Elevated paternal glucocorticoid exposure alters the small noncoding RNA profile in sperm and modifies anxiety and depressive phenotypes in the offspring. *Translational psychiatry* **6**, e837, doi:10.1038/tp.2016.109 (2016).

17 Chen, Q. *et al.* Sperm tsRNAs contribute to intergenerational inheritance of an acquired metabolic disorder. *Science (New York, N.Y.)* **351**, 397-400, doi:10.1126/science.aad7977 (2016).

18 Sharma, U. *et al.* Biogenesis and function of tRNA fragments during sperm maturation and fertilization in mammals. *Science (New York, N.Y.)* **351**, 391-396, doi:10.1126/science.aad6780

(2016).

19 Gao, W. *et al.* Paternal heroin self-administration in rats increases drug-seeking behavior in male offspring via miR-19b downregulation in the nucleus accumbens. *Neuropsychopharmacology : official publication of the American College of Neuropsychopharmacology* **50**, 1027-1038, doi:10.1038/s41386-025-02081-8 (2025).

20 Sanchez-Rangel, E. & Inzucchi, S. E. Metformin: clinical use in type 2 diabetes. *Diabetologia* **60**, 1586-1593, doi:10.1007/s00125-017-4336-x (2017).

21 Zhang, S. Y. *et al.* Metformin triggers a kidney GDF15-dependent area postrema axis to regulate food intake and body weight. *Cell metabolism* **35**, 875-886.e875, doi:10.1016/j.cmet.2023.03.014 (2023).

22 Liu, J., Aylor, K. W., Chai, W., Barrett, E. J. & Liu, Z. Metformin prevents endothelial oxidative stress and microvascular insulin resistance during obesity development in male rats. *American journal of physiology. Endocrinology and metabolism* **322**, E293-e306, doi:10.1152/ajpendo.00240.2021 (2022).

23 Ekperikpe, U. S. *et al.* Metformin reduces insulin resistance and attenuates progressive renal injury in prepubertal obese Dahl salt-sensitive rats. *American journal of physiology. Renal physiology* **325**, F363-f376, doi:10.1152/ajprenal.00078.2023 (2023).

24 Xi, Y. & Li, W. BSMAP: whole genome bisulfite sequence MAPping program. *BMC bioinformatics* **10**, 232, doi:10.1186/1471-2105-10-232 (2009).

25 Jühling, F. *et al.* metilene: fast and sensitive calling of differentially methylated regions from bisulfite sequencing data. *Genome research* **26**, 256-262, doi:10.1101/gr.196394.115 (2016).

26 Love, M. I., Huber, W. & Anders, S. Moderated estimation of fold change and dispersion for RNA-seq data with DESeq2. *Genome biology* **15**, 550, doi:10.1186/s13059-014-0550-8 (2014).

27 Yu, G., Wang, L. G., Han, Y. & He, Q. Y. clusterProfiler: an R package for comparing biological themes among gene clusters. *Omics : a journal of integrative biology* **16**, 284-287, doi:10.1089/omi.2011.0118 (2012).

28 Feng, C. *et al.* KnockTF 2.0: a comprehensive gene expression profile database with knockdown/knockout of transcription (co-)factors in multiple species. *Nucleic acids research* **52**, D183-d193, doi:10.1093/nar/gkad1016 (2024).

29 Huang, T. *et al.* Adipocyte-derived kynurenine promotes obesity and insulin resistance by activating the AhR/STAT3/IL-6 signaling. *Nature communications* **13**, 3489, doi:10.1038/s41467-022-31126-5 (2022).

30 Hindi, L., McMillan, J. D., Afroze, D., Hindi, S. M. & Kumar, A. Isolation, Culturing, and Differentiation of Primary Myoblasts from Skeletal Muscle of Adult Mice. *Bio-protocol* **7**, doi:10.21769/BioProtoc.2248 (2017).
